# Supplementary material for: Population structure across scales facilitates coexistence and spatial heterogeneity of antibiotic-resistant infections
Source: PLoS Comput Biol. 2020 Jul 6;16(7):e1008010. doi: 10.1371/journal.pcbi.1008010 (PMC7365476; doi:10.1371/journal.pcbi.1008010)
Supplement: S1 Text — Additional information on model derivation and analysis, equations for models with alternative drug action, and computational methods. (PDF) [file pcbi.1008010.s001.pdf]

SUPPLEMENTARY METHODS

Population structure across scales facilitates coexistence and spatial heterogeneity of antibiotic-resistant infections

Madison S. Krieger[1,*], Carson E. Denison[1,†], Thayer L. Anderson[1,†], Martin A. Nowak[1], and Alison L. Hill[1]

[1]Department of Organismic & Evolutionary Biology, Harvard University, Cambridge Massachusetts, MA, USA
[*]mkrieger@fas.harvard.edu
[†]These authors contributed equally to this work

Analytic results for well-mixed single deme with treatment

The dynamics of competition between drug-sensitive and drug-resistant strains of infection in an infinite, well-mixed population where a fraction f of individuals receive treatment if infected is described by Eq. 1. This system of equations tracks the *proportion* of the population in each treatment and infection category (infected with the wild type and untreated (w_u), infected with the wild type and treated (w_t), infected with the resistant strain and untreated (r_u), and infected with the resistant strain and treated (r_t)). These equations were developed by first writing down the dynamics of the *number* of individuals in each state, including uninfected (susceptible):

$$\begin{aligned}
\dot{S_u} &= -k\left(W_u + (1-\epsilon)W_t + (1-c)(R_u+R_t)\right)S_u + g(W_u+R_u), \\
\dot{W_u} &= k\left(W_u + (1-\epsilon)W_t\right)S_u - gW_u, \\
\dot{R_u} &= k(1-c)(R_u+R_t)S_u - gR_u, \\
\dot{S_t} &= -k\left(W_u + (1-\epsilon)W_t + (1-c)(R_u+R_t)\right)S_t + g(W_t+R_t), \\
\dot{W_t} &= k(W_u + (1-\epsilon)W_t)S_t - gW_t, \\
\dot{R_t} &= k(1-c)(R_u+R_t)S_t - gR_t.
\end{aligned} \tag{S.1}$$

The transmission rate k represents the rate at which each infected individual contacts and infects each susceptible individual. Although we use the terms S_t and S_u to track susceptible individuals who are "treated" or "untreated", our model is agnostic as to whether these uninfected individuals actually receive drug, or if these markers simply indicate if these individuals will (or will not) receive treatment *when infected*. While here we assume a pre-determined fraction f of the total population will receive treatment if infected, similar models could be constructed assuming the decision to receive treatment is made instantaneously upon infection with probability f, or that there is some rate of receiving treatment, such that a fraction f will receive treatment before recovery. The analysis of these models gives identical steady state results.

Because in our model the total population size N, as well as the fraction of individuals who will (or will not) receive treatment if infected, are both constant, we can remove the variables for susceptible individuals using $S_u = N(1-f) - W_u - R_u$ and $S_t = Nf - W_t - R_t$. To further reduce the variables in this system and remove any dependence on total population size, we let $\kappa = kN$ and $x_i = X_i/N$ (the fraction of individuals instead of the total number, for $X = W, R$ and $i = u, t$), which then leads to Eq. 1. By keeping $\kappa = kN$ as the

constant parameter, we are implicitly assuming that the total rate at which a single individual can contact and infect others when the population is completely susceptible (kN) is independent of the population size. This is a reasonable assumption for many infections which require some type of active contact, and here allows us to reduce the number of parameters required. None of our results depend on this scaling, and the effect of changing population size is equivalent to changing κ in this deterministic model.

There are three possible equilibria of Eq. 1. In each of these equilibria, there is no possible coexistence of infections. These equilibria are as follows:

1. No infection survives

$$w_u = r_u = w_t = r_t = 0 \tag{S.2}$$

This uninfected equilibrium is only stable when

$$\begin{aligned} R_0^w &= \frac{\kappa(1 - f\epsilon)}{g} < 1 \\ R_0^r &= \frac{\kappa(1 - c)}{g} < 1 \end{aligned} \tag{S.3}$$

2. Only wild-type (drug-sensitive) strain survives

$$\begin{aligned} w_u &= (1 - f)\left(1 - \frac{1}{R_0^w}\right) \\ r_u &= 0 \\ w_t &= f\left(1 - \frac{1}{R_0^w}\right) \\ r_t &= 0 \end{aligned} \tag{S.4}$$

This infected equilibrium is stable when

$$\begin{aligned} R_0^w &> 1 \\ R_0^r &< R_0^w \end{aligned} \tag{S.5}$$

where the later condition implies $c > f\epsilon$.

3. Only drug-resistant strain survives

$$\begin{aligned} w_u &= 0 \\ r_u &= (1 - f)\left(1 - \frac{1}{R_0^r}\right) \\ w_t &= 0 \\ r_t &= f\left(1 - \frac{1}{R_0^r}\right) \end{aligned} \tag{S.6}$$

This infected equilibrium is stable when

$$\begin{aligned} R_0^r &> 1 \\ R_0^w &< R_0^r \end{aligned} \tag{S.7}$$

where the former condition implies $c < f\epsilon$.

Analytic results for two connected subpopulations

In an effort to be more general than in the main text, we consider the case of a population of total size N divided into two demes, with a fraction f of the total population is contained in the drug-treated deme. The system describing the *number* of individuals in each state can then be written as:

$$\begin{aligned}
\dot{S}_u &= -(kW_u + b(1-\epsilon)W_t + (1-c)(kR_u + bR_t))S_u + g(W_u + R_u), \\
\dot{W}_u &= (kW_u + b(1-\epsilon)W_t)S_u - gW_u, \\
\dot{R}_u &= (1-c)(kR_u + bR_t)S_u - gR_u, \\
\dot{S}_t &= -S_t(bW_u + k(1-\epsilon)W_t + (1-c)(bR_u + kR_t)) + g(W_t + R_t), \\
\dot{W}_t &= (bW_u + k(1-\epsilon)W_t)S_t - gW_t, \\
\dot{R}_t &= (1-c)(bR_u + kR_t)S_t - gR_t.
\end{aligned} \tag{S.8}$$

Since the size of each deme is fixed, we can remove the variables for susceptible individuals using $S_u = N(1-f) - W_u - R_u$ and $S_t = Nf - W_t - R_t$. To create a nondimensional version of the system, we instead track the *fraction* of individuals in each deme who have a particular infection status, so $w_u = W_u/(N(1-f))$, $R_u = R_u/(N(1-f))$, $w_t = W_t/(Nf)$, $R_t = R_t/(Nf)$, and $\kappa = kN$ and $\beta = bN$, which gives the altered system:

$$\begin{aligned}
\dot{w_u} &= (\kappa(1-f)w_u + \beta f(1-\epsilon)w_t)(1 - w_u - r_u) - gw_u, \\
\dot{r_u} &= (1-c)(\kappa(1-f)r_u + \beta f r_t)(1 - w_u - r_u) - gr_u, \\
\dot{w_t} &= (\beta(1-f)w_u + \kappa f(1-\epsilon)w_t)(1 - w_t - r_t) - gw_t, \\
\dot{r_t} &= (1-c)(\beta(1-f)r_u + \kappa f r_t)(1 - w_t - r_t) - gr_t.
\end{aligned} \tag{S.9}$$

Using the next-generation technique [**?**], we arrive at the following values for the basic reproductive ratios,

$$\begin{aligned}
R_0^w &= \frac{1}{2}\frac{\kappa}{g}\left((1-f\epsilon) + \sqrt{(1-f\epsilon)^2 - 4\left(1 - \frac{\beta^2}{\kappa^2}\right) f(1-f)(1-\epsilon)}\right), \\
R_0^r &= \frac{1}{2}\frac{\kappa}{g}(1-c)\left(1 + \sqrt{1 - 4\left(1 - \frac{\beta^2}{\kappa^2}\right) f(1-f)}\right).
\end{aligned} \tag{S.10}$$

In general, this system has nine equilibria. However, only one of these is stable and physically realizable (e.g. having between 0% and 100% of individuals infected) for any given parameter set. These are the values that are plotted in the main text. These outcomes fall into one of two qualitative categories. Either one strain drives the other to competitive extinction (true in Fig 3 for $c > 0.4$ and $c < 0.06$, for instance) or both strains exist in both demes simultaneously. The boundaries separating this region from the competitive-exclusion region are $R_0^r = R_0^w$, the lower bound when viewed as a function of the cost of resistance c, and the curve which solves $\beta(1-f)w_u^* + \kappa f(1-\epsilon)w_t^* = \beta(1-f)(1-c)r_u^* + \kappa f(1-c)r_t^*$, where $[w_u^*, w_t^*, r_u^*, r_t^*]$ denote the equilibrium values. This second curve, in words, is where the drug-treated population is infected at the same rate by both drug-resistant and drug-sensitive strains.

For the parameters chosen here and used throughout the paper, it is impossible to have $R_0^w < 1$. For sufficiently high c, we can have $R_0^r < 1$. However, this line in parameter space lies deep within the region where the population is infected with the wild-type strain only.

Derivation of a multi-deme model

Here we derive the general case for the spread of wild-type and resistant strains of infection in a population consisting of multiple connected demes (with connectivity described by adjacency matrix Δ), where demes may have different sizes (D_i) and different treatment levels (f_i). The dynamics of infection are described by the following system of equations

$$
\begin{aligned}
\dot{S}_{ui} =& -\left(\left[k(W_{ui}+(1-\epsilon)W_{ti})+b\sum_j \Delta_{ji}(W_{uj}+(1-\epsilon)W_{tj})\right]+(1-c)\left[k(R_{uj}+R_{tj})+b\sum_j \Delta_{ji}(R_{ui}+R_{ti})\right]\right)S_{ui}\\
&+g(W_{ui}+R_{ui})\\
\dot{S}_{ti} =& -\left(\left[k(W_{ui}+(1-\epsilon)W_{ti})+b(\sum_j \Delta_{ji}(W_{uj}+(1-\epsilon)W_{tj}))\right]+(1-c)\left[k(R_{uj}+R_{tj})+b\sum_j \Delta_{ji}(R_{ui}+R_{ti})\right]\right)S_{ti}\\
&+g(W_{ti}+R_{ti})\\
\dot{W}_{ui} =& \left[k(W_{ui}+(1-\epsilon)W_{ti})+b\sum_j \Delta_{ji}(W_{uj}+(1-\epsilon)W_{tj})\right]S_{ui}-gW_{ui}\\
\dot{W}_{ti} =& \left[k(W_{ui}+(1-\epsilon)W_{ti})+b\sum_j \Delta_{ji}(W_{uj}+(1-\epsilon)W_{tj})\right]S_{ti}-gW_{ti}\\
\dot{R}_{ui} =& (1-c)\left[k(R_{uj}+R_{tj})+b\sum_j \Delta_{ji}(R_{ui}+R_{ti})\right]S_{ui}-gR_{ui}\\
\dot{R}_{ti} =& (1-c)\left[k(R_{uj}+R_{tj})+b\sum_j \Delta_{ji}(R_{ui}+R_{ti})\right]S_{ti}-gR_{ti}
\end{aligned}
\tag{S.11}
$$

where X_{it} represents the total number of individuals in deme i infected with strain X (or susceptible, S) who will be treated if infected, whereas X_{iu} is those who won't be treated if infected.

Since the population size D_i of each deme is constant and we assume it is predetermined who will get treatment *if* infected, we can remove the equations for the susceptible individuals using $D_{it} = f_i D_i = S_{it} + W_{it} + R_{it}$ and $D_{iu} = (1-f_i)D_i = S_{iu} + W_{iu} + R_{iu}$.

To derive a dimensionless form the the equations, we let $x_i = X_i/D_i$, and define $\sigma_i = D_i/\bar{D}$ where $\bar{D}$ is the average deme size. We define $\kappa = k\bar{D}$ and $\beta = b\bar{D}$, to derive the simplified and scaled equations

$$
\begin{aligned}
\dot{w}_{ui} &= \left[\kappa\sigma_i(w_{ui}+(1-\epsilon)w_{ti})+\beta\sum_j \sigma_j\Delta_{ji}(w_{uj}+(1-\epsilon)w_{tj})\right](1-f_i-w_{ui}-r_{ui})-gw_{ui}\\
\dot{w}_{ti} &= \left[\kappa\sigma_i(w_{ui}+(1-\epsilon)w_{ti})+\beta\sum_j \sigma_j\Delta_{ji}(w_{uj}+(1-\epsilon)w_{tj})\right](f_i-w_{ti}-r_{ti})-gw_{ti}\\
\dot{r}_{ui} &= (1-c)\left[\kappa\sigma_i(r_{ui}+r_{ti})+\beta\sum_j \sigma_j\Delta_{ji}(r_{uj}+r_{tj})\right](1-f_i-w_{ui}-r_{ui})-gr_{ui}\\
\dot{r}_{ti} &= (1-c)\left[\kappa\sigma_i(r_{ui}+r_{ti})+\beta\sum_j \sigma_j\Delta_{ji}(r_{uj}+r_{tj})\right](f_i-w_{ti}-r_{ti})-gr_{ti}
\end{aligned}
\tag{S.12}
$$

In the limit of either 0% or 100% treatment in a deme, this system reduces to

$$\dot{w}_i = \left[\kappa\sigma_i(1-\epsilon T_i)w_i + \beta\sum_j \sigma_j\Delta_{ji}(1-\epsilon T_j)w_j\right](1-w_i-r_i) - gw_i,$$
$$\dot{r}_i = (1-c)\left[\kappa\sigma_i r_i + \beta\sum_j \sigma_j\Delta_{ji}r_j\right](1-w_i-r_i) - gr_i. \quad \text{(S.13)}$$

In the limit of both constant deme size and 0% or 100% treatment in a deme, this system reduces to Eq. 3.

The apparent absence of multiple equilibria

Although the generic system of equations modeled by Eq. 3 admits multiple equilibria, we actually never observed any dynamic phenomena other than a steady convergence to a single stable fixed point, even when initial conditions were wildly different from that considered in the main text. It is not uncommon for infection models to admit equilibria which are physically unrealizable, i.e., taking values outside the unit simplex — it may therefore be the case that any other stable equilibria are simply unphysical.

However, to comprehensively demonstrate this fact — either theoretically or numerically — is outside the scope of the current work. To prove such a result theoretically would necessarily draw on ideas of nonlinear algebra or algebraic geometry, such as intersection theory, which are outside the present scope; to demonstrate numerically that for all parameter sets and graph structures there is only one stable equilibrium is computationally infeasible, even on graphs of a given small size.

To illustrate the idea that there seems to be only one stable equilibrium per system, we fixed an exact distribution of treatment with $\rho = 0.35$ to be used on every random regular graph used in the main text. This means that, since all nodes in all graphs are labelled (and the random regular graphs in the main text have twenty demes each), seven labelled demes (here 1, 3, 8, 11, 15, 19, 20) were drug-treated irrespective of graph structure. We then performed, for all 30 graphs, 100 trials with uniformly random initial conditions over every deme, i.e., every deme can start with any possible arrangement of wild-type and resistant infectives (so long as the sum of the two does not exceed 1). We then compared the equilibria seen with the equilibria found for the initial condition used in the main body of the text.

There are many possible metrics which would illustrate the relative closeness of all the equilibria seen. In S17 Fig we have elected to plot the maximum matrix norm (the 2-norm) of the difference between the equilibrium seen in any of the 100 trials and the equilibrium from the main text. That is to say, all of the trial equilibria differ in norm from the main-text equilibrium by an amount not more than that plotted in the figure for a given graph structure. The parameters used were our default: κ=0.25, β=0.05/day, g=0.1/day, ϵ=0.9, c=0.2.

In all of these cases, the maximum norm deviation is extremely small. This could be due to two possible reasons. The first is due simply to aggregated numerical error from the many steps involved of a) calculating the equilibrium (which involves a threshold of stopping when the time-derivatives in all demes fall below a certain threshold, which can allow for some very small freedom in what an "equilibrium" is in a given trial), b) calculating the matrix norm (which amplifies these small errors). The second explanation is that while multiple stable equilibria may exist, they are extremely close together, with almost clinically-unmeasurable differences in the rates of wild-type and resistant infections within a given deme in a given community.

Model with effects of treatment on recovery rates

To demonstrate the robustness of the results described in the main text, we explored a second model in which the drug, rather than inhibiting the spread of the infection, instead speeds up recovery. The effect of treatment is to increase the rate of recovery from g to $g+\tau$. In this model formulation a perfectly efficacious drug would correspond to $\tau \to \infty$ (instantaneous clearance of infection). A finite value of τ takes into

account the fact that it takes some time for an infected individual to seek a clinician, obtain a prescription for drug, and clear the infection as a result of treatment.

This model matches qualitatively with that discussed in the main text, in that in reproduces the spatial motifs in the same way. The two models are also quantitatively quite similar for reasonably-akin parameter sets. Here we reproduce the main results for this alternative model.

A single well-mixed deme

Here, the system can be written as

$$\begin{aligned} \dot{w_u} &= \kappa\,(w_u + w_t)\,((1-f) - w_u - r_u) - g w_u, \\ \dot{r_u} &= \kappa(1-c)(r_u + r_t)((1-f) - w_u - r_u) - g r_u, \\ \dot{w_t} &= \kappa(w_u + w_t)(f - w_t - r_t) - (g+\tau) w_t, \\ \dot{r_t} &= \kappa(1-c)(r_u + r_t)(f - w_t - r_t) - g r_t. \end{aligned} \tag{S.14}$$

The basic reproductive ratio R_0 for each strain alone is

$$\begin{aligned} R_0^w &= \frac{k}{g}\left(1 - \frac{f\tau}{g+\tau}\right), \\ R_0^r &= \frac{\kappa(1-c)}{g}. \end{aligned} \tag{S.15}$$

For the purpose of comparing this model, the value of τ for which R_0^w in the main model (with our baseline $\epsilon = 0.9$) equals R_0^w in this alternative model is $\tau = 0.9$. This system admits only three stable, physical (having fractions of treated individuals between 0 and 1) solutions: one in which all infections die out ($R_0^w < 1$, $R_0^r < 1$), one in which only wild-type infections persist ($R_0^w > 1$, $R_0^r < R_0^w$) and one in which only resistant infection persists ($R_0^r > 1, R_0^w < R_0^r$). There is no possibility for coexistence. The condition for resistance to persist (instead of wild type)is $c < \frac{f\tau}{g+\tau}$.

Two connected demes

In this model, the equivalent of Eq. S.9 can be written as

$$\begin{aligned} \dot{w_u} &= (\kappa(1-f)w_u + \beta f w_t)(1 - w_u - r_u) - g w_u, \\ \dot{r_u} &= (1-c)(\kappa(1-f)r_u + \beta f r_t)(1 - w_u - r_u) - g r_u, \\ \dot{w_t} &= (\beta(1-f)w_u + \kappa f w_t)(1 - w_t - r_t) - (g+\tau) w_t, \\ \dot{r_t} &= (1-c)(\beta(1-f)r_u + \kappa f r_t)(1 - w_t - r_t) - g r_t. \end{aligned} \tag{S.16}$$

Using the next-generation technique, we were able to derive the values of R_0 in a two-deme system for the alternative model, as an analog to Eq. S.10:

$$\begin{aligned} R_0^w &= \frac{1}{2}\frac{\kappa}{g}\left(\left(1 - \frac{f\tau}{g+\tau}\right) + \sqrt{\left(1 - \frac{f\tau}{g+\tau}\right)^2 - 4\left(1 - \frac{\beta^2}{\kappa^2}\right) f(1-f)\frac{g}{g+\tau}}\right), \\ R_0^r &= \frac{1}{2}\frac{\kappa}{g}(1-c)\left(1 + \sqrt{1 - 4\left(1 - \frac{\beta^2}{\kappa^2}\right) f(1-f)}\right). \end{aligned} \tag{S.17}$$

The expressions are slightly more complicated, but the curves generated are similar to the main text. S7 Fig shows the analog of Fig 3. As in the main text, here the boundaries of the coexistence region are the curves $R_0^w = R_0^r$ (lower boundary) and the implicit curve where more wild-type than resistant infected individuals are produced per unit time in the drug-treated deme (upper boundary).

Multi-deme population

The scaled version of the multi-deme model presented in Eq. 3 for the case of treatment acting on the recovery rates is

$$\begin{aligned} \dot{w}_i &= \left[\kappa w_i + \beta \sum_j \Delta_{ji} w_j\right] (1 - w_i - r_i) - (g + T_i \tau) w_i, \\ \dot{r}_i &= (1 - c)\kappa \left[r_i + \beta \sum_j \Delta_{ji} r_j\right] (1 - w_i - r_i) - g r_i. \end{aligned} \tag{S.18}$$
