# Supplementary figures and images for: Population structure across scales facilitates coexistence and spatial heterogeneity of antibiotic-resistant infections
Source: PLoS Comput Biol. 2020 Jul 6;16(7):e1008010. doi: 10.1371/journal.pcbi.1008010 (PMC7365476; doi:10.1371/journal.pcbi.1008010)

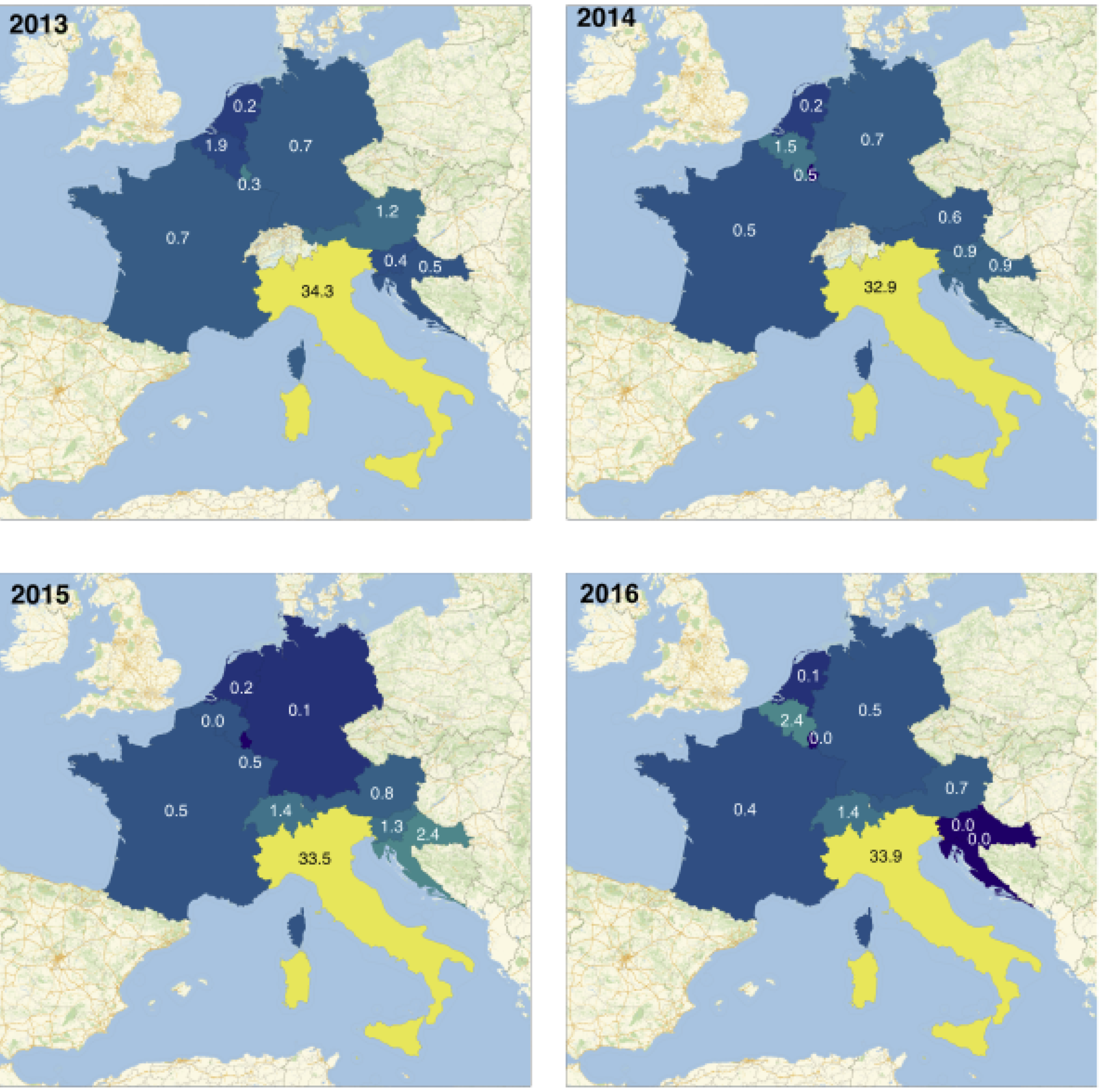

Supplement: S1 Fig — Percent of Klebsiella pneumoniae isolates resistant to carbapenems in Austria, Belgium, Croatia, France, Germany, Italy, Luxembourg, and Slovenia from 2013-2016 as reported in the ECDC’s European Antibiotic Resistance Surveillance Network [13, 14] and Switzerland as reported by the Swiss Centre for Antibiotic Resistance from 2015-2016. Each country is labeled with the resistance level (%). The year-to-year deviation in Italy from the average value shown in Fig 1 is less than 1% for all years. For all other countries, the frequency of resistance never reaches even 1/10 of the average value seen in Italy. (TIFF) [file pcbi.1008010.s002.tiff]

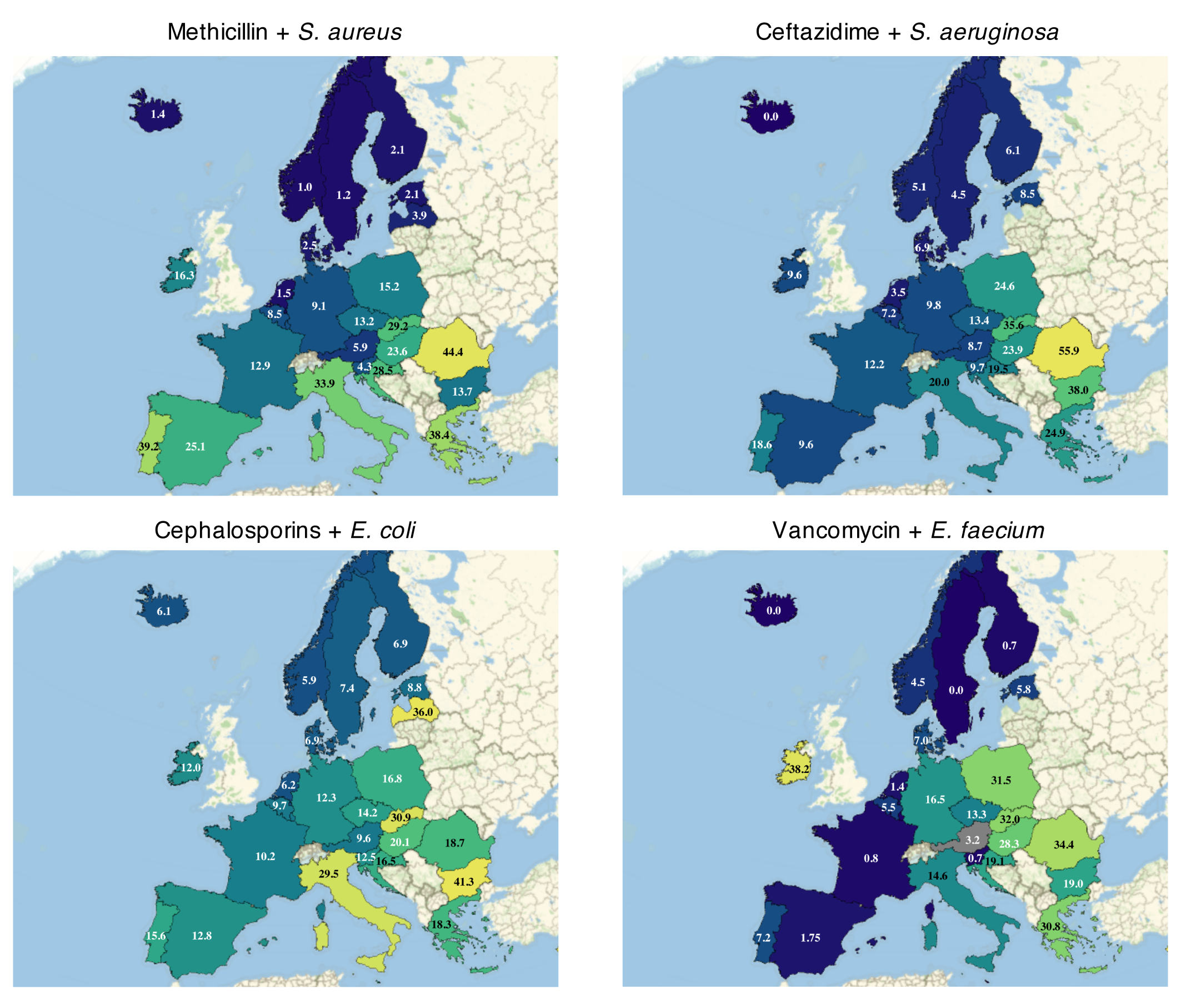

Supplement: S2 Fig — Percent of isolates resistant to particular antibiotics as reported in the ECDC’s European Antibiotic Resistance Surveillance Network [13, 14] in 2017. Each country is labeled with the resistance level (%). Four example bug-drug pairs are shown: Staphylococcus aureaus + methicillin, Pseudomonas aeruginosa + ceftazadime, Eschericia coli + cephalosporins, Enterococcus faecium + vancomycin. (TIF) [file pcbi.1008010.s003.tif]

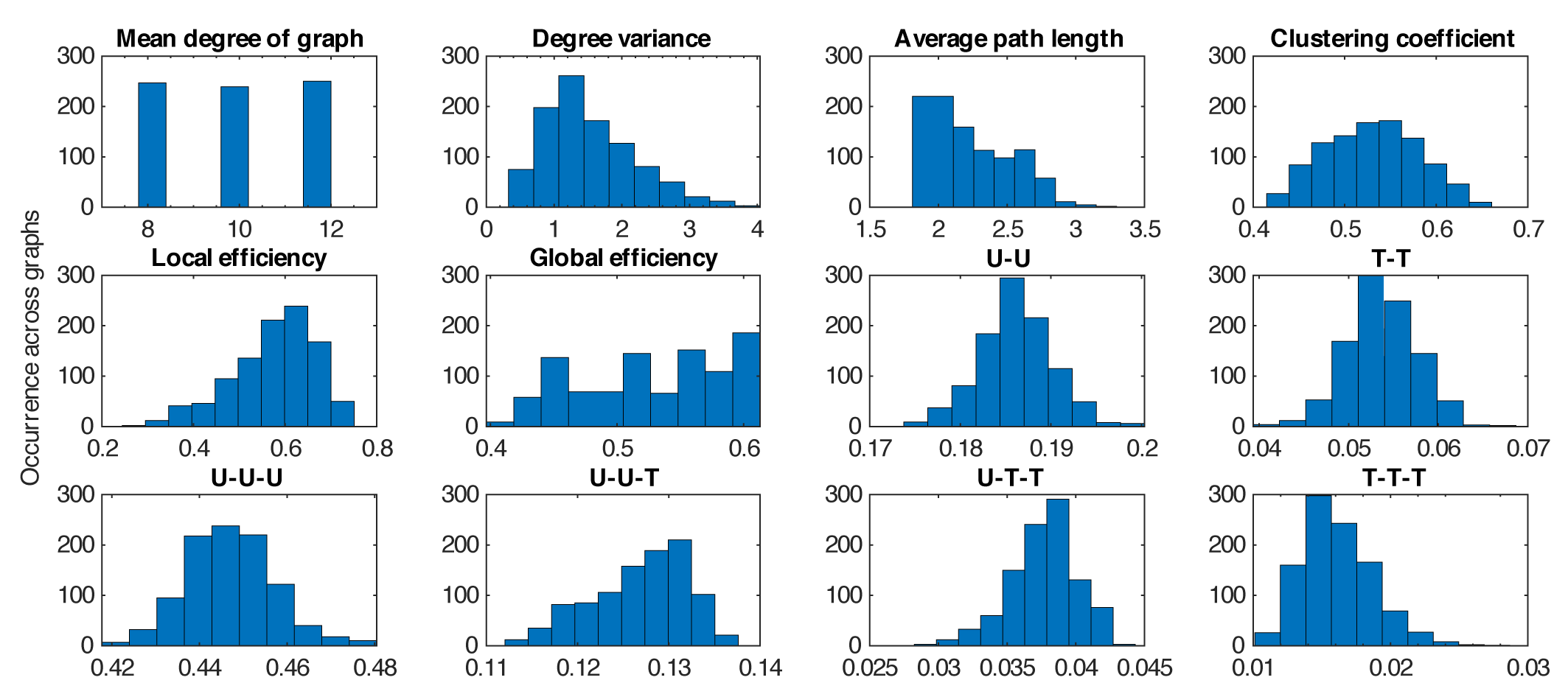

Supplement: S3 Fig — Populations were created as random networks of 50 demes using a variant of the Watts-Strogatz algorithm. Treatment was randomly assigned to a portion ρ of demes so that an overall desired fraction treated was achieved (here, ρ = 0.24). Distributions show properties for 1000 such networks. The first six properties are intrinsic to the network structure, while the latter six describe how treatment is allocated over the network. Quantities of the form X-Y give the proportion of all pairs of connected demes in which one deme has treatment status X and one has treatment status Y (U = untreated, T = treated). Quantities of the form X-Y-Z give the same information for triples of connected demes (order ignored). A description of the methods for creating the networks and for calculating each property is given in the Methods. (TIF) [file pcbi.1008010.s004.tif]

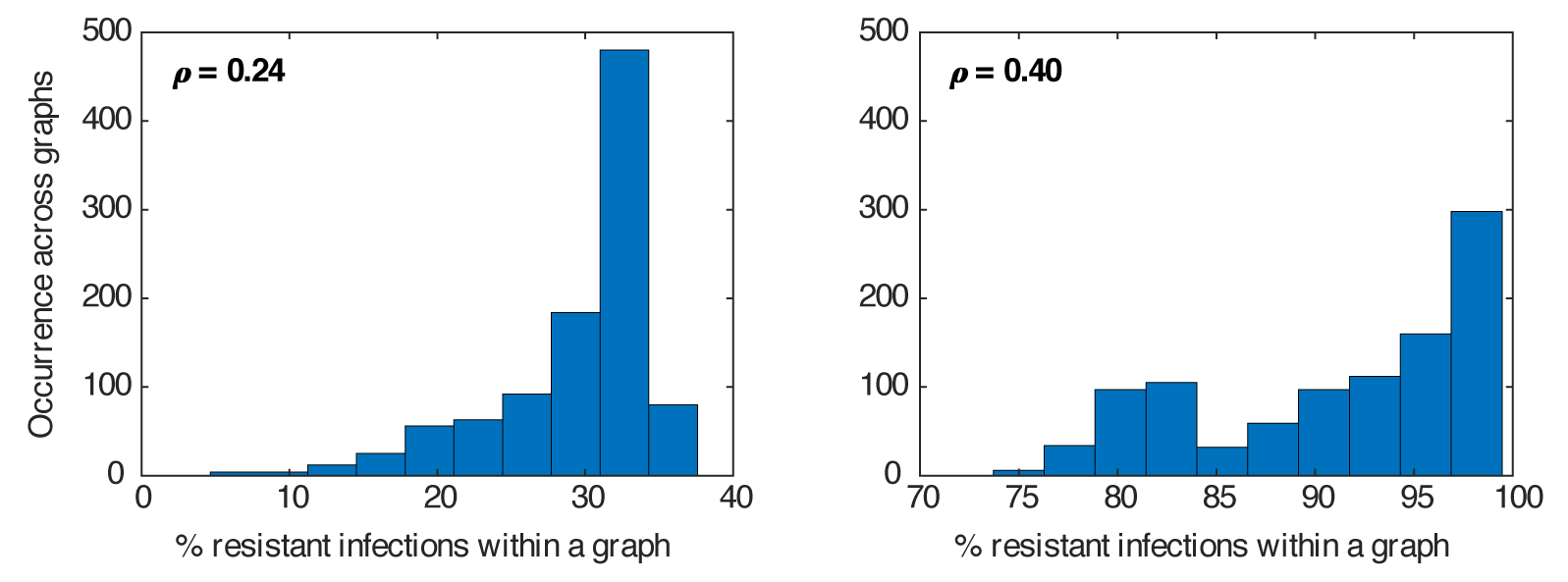

Supplement: S4 Fig — Populations were created as random networks of 50 demes using a variant of the Watts-Strogatz algorithm. Treatment was randomly assigned to a portion ρ of demes so that an overall desired fraction treated was achieved (on the left ρ = 0.24, and on the right ρ = 0.4). Infection dynamics were simulated (Eq (3)) until an equilibrium was reached, at which the proportion of all infections with the drug-resistant strain was recorded. Distributions show results for 1000 such simulations each with a unique random network and treatment allocation. Parameters used were κ = 0.25/day, β = 0.05/day, g = 0.1/day, ϵ = 0.9, c = 0.2. (TIF) [file pcbi.1008010.s005.tif]

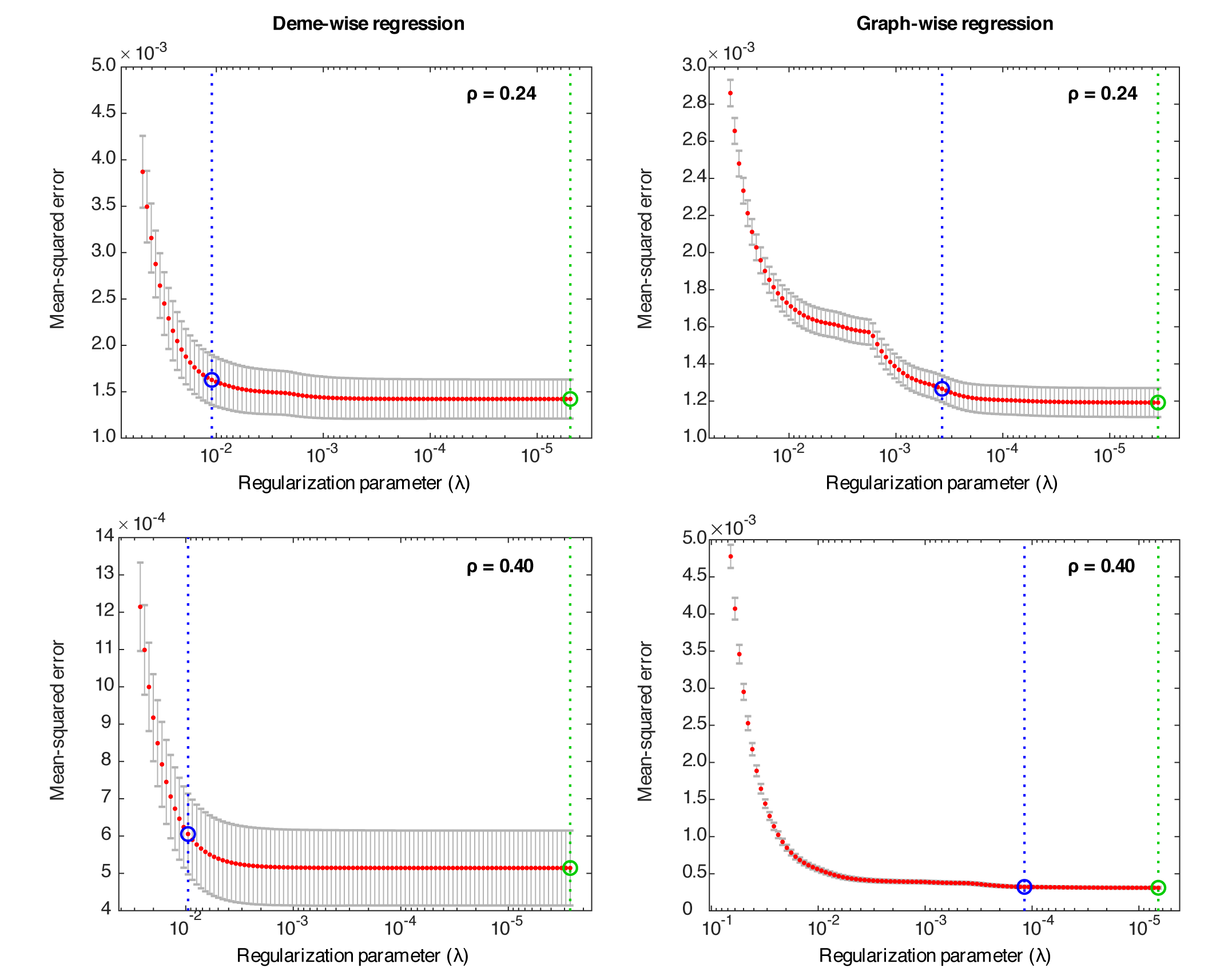

Supplement: S5 Fig — Mean-squared error of model predictions vs sub-sample of 1/5 of data after model fitting to 4/5 of data, as a function of the internal parameter penalizing the L1 norm for fitted coefficients (λ). Red dots show the mean-squared error of the model, with grey bars showing the standard error of the mean. The green circle/line indicates the value of λ that gives the minimum cross-validation error, whereas the blue circle/line locates the λ value where the mean-squared error is one standard error above that of the λ value with minimum cross-validation error. (TIF) [file pcbi.1008010.s006.tif]

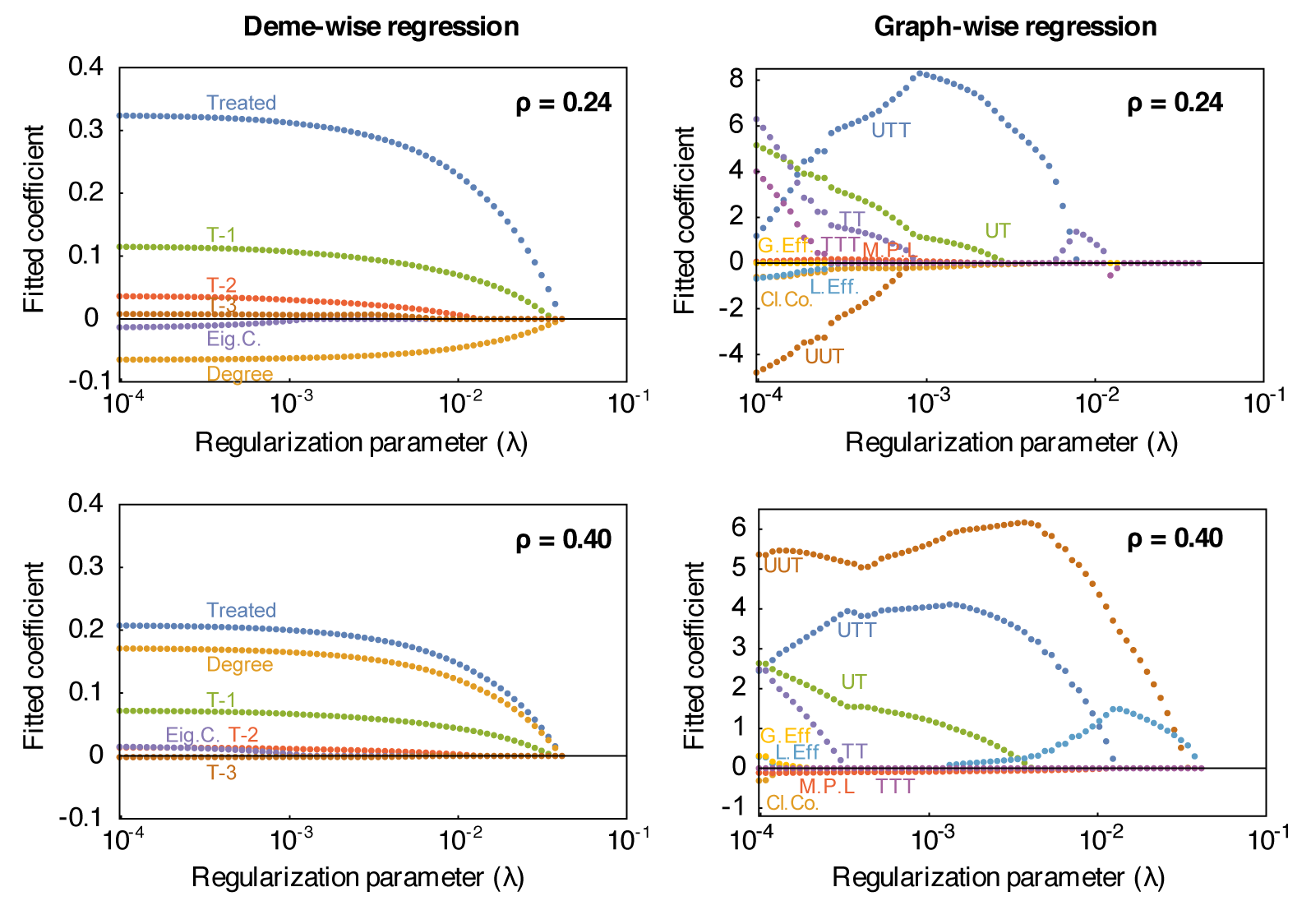

Supplement: S6 Fig — Plots show the regression coefficients obtained as a function of the internal constraint on L1 norm of coefficients (“regularization parameter”, λ). Each curve is a different predictor variable (described in Table 1). Analysis were separately run to predict deme-level resistance from deme-level properties (left side) or population-level resistance from graph-level properties (right side). Each analysis was conducted for two different levels of drug coverage in the population (either a fraction ρ = 0.24 or ρ = 0.4 of demes treated). The labels on the curves match the names of the predictors in Table 1. (TIF) [file pcbi.1008010.s007.tif]

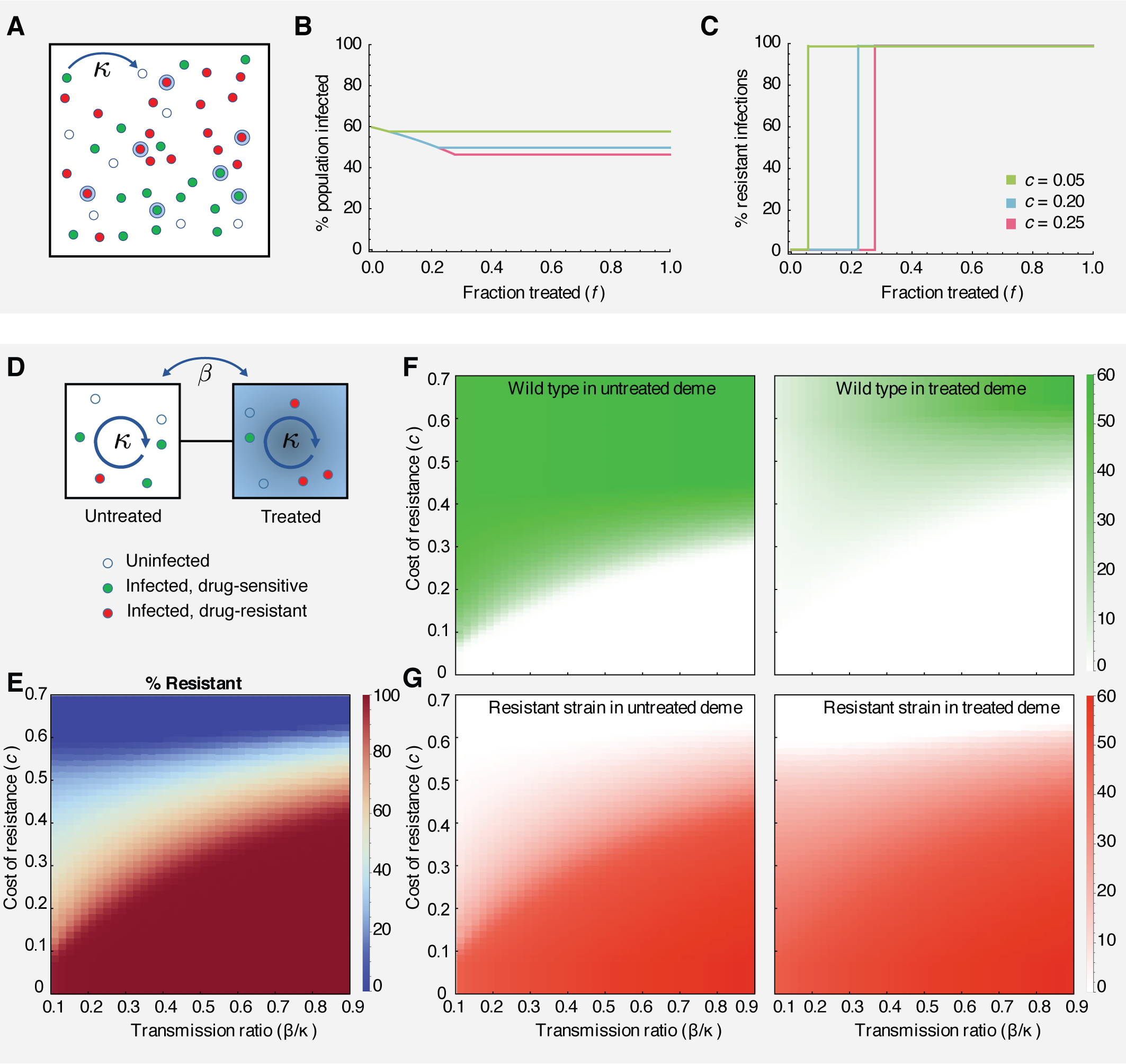

Supplement: S7 Fig — A) A population of individuals in a single well-mixed deme, in which a fraction f will receive drug treatment when infected (blue haloes). Individuals may be uninfected (hollow blue circles), or infected with either the wild-type (green circles) or drug-resistant (red circles) strain. B) The total prevalence of infection (wild-type + drug-resistant) as a function of the fraction of treated individuals (f) for different costs of resistance (c). C) The % of infections that are drug-resistant as a function of the fraction of treated individuals (f) for different parameters. Infection switches between 0% and 100% resistant when f=c(g+τ)τ. Coexistence never occurs. D) Schematic of a two-deme population (left-untreated, right-treated) and the two strains (green-wild type, red-resistant) considered in the model. E-G) Each panel shows the infection level (shading) as a function of the relative connectivity between demes (β/κ) and the cost of resistance (c). E) The % of all infections that are drug-resistant strain across the entire population. F) The % of individuals in each deme who are infected with the wild type strain. G) The % of individuals in each deme who are infected with the resistant strain. For all results, the transmission rate is κ = 0.25/day, the recovery rate is g = 0.1/day, and the increase in recovery rate due to treatment is treatment efficacy is τ = 0.9/day. (TIF) [file pcbi.1008010.s008.tif]

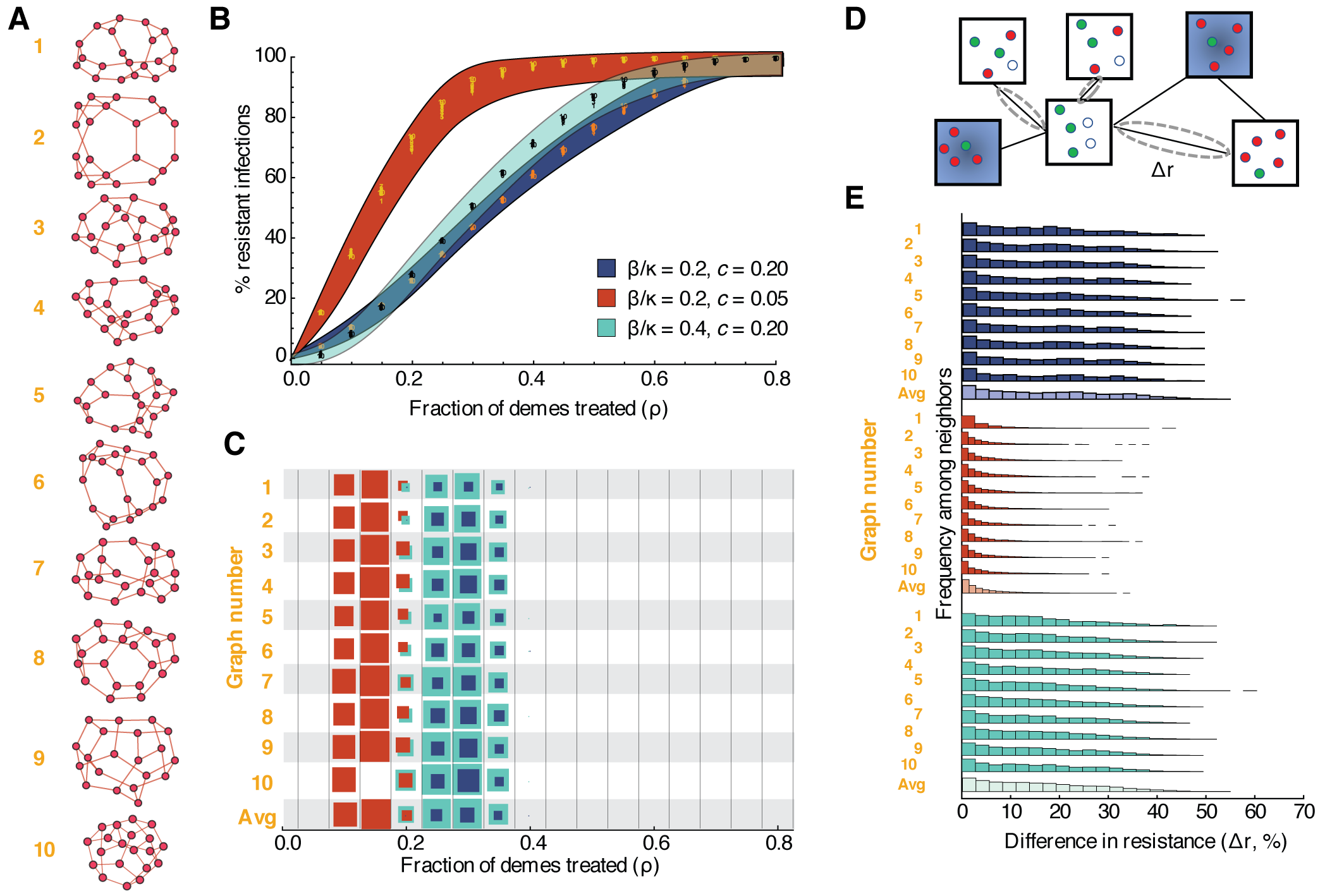

Supplement: S8 Fig — A) Randomly generated population structures on which infection was simulated. Each node represents a deme (a well-mixed sub-population of individuals), and each edge indicates that infection can spread in either direction between those two demes. Ten example populations were selected out of 1000 total simulated, each with twenty demes randomly connected to three neighbors each, to represent a broad range of outcomes. B) Fraction of infections that are resistant in the entire population (y-axis) versus fraction of demes treated, ρ (x-axis). Each color represents a different parameter set (blue background—baseline, red background—lower cost of resistance, teal background—more between-deme connectivity). Numbers show data points for the ten example populations. The colored envelope is created by shading between sigmoidal curves that encompass all the data. C) For each population structure shown (y-axis) and each treatment level (x-axis), the proportion of simulations that resulted in robust coexistence between drug-sensitive and drug-resistant strains is shown (by the colored area of the box). Robust coexistence was defined as at least 80% of demes supporting both strains at frequencies above 10%. D) Differences in resistance levels (% of all infections that are with the drug resistant-strain) are measured between all pairs of directly-connected untreated demes. E) Histograms showing the distribution of pairwise differences in resistance for a given population structure. Lighter shaded histograms combine results from all population graphs. All simulations used kinetic parameters κ = 0.25, g = 0.1, and τ = 0.9, and pooled results from 100 simulations with different random allocation of treatment across demes. Pairwise differences were calculated with 30% treatment. (TIF) [file pcbi.1008010.s009.tif]

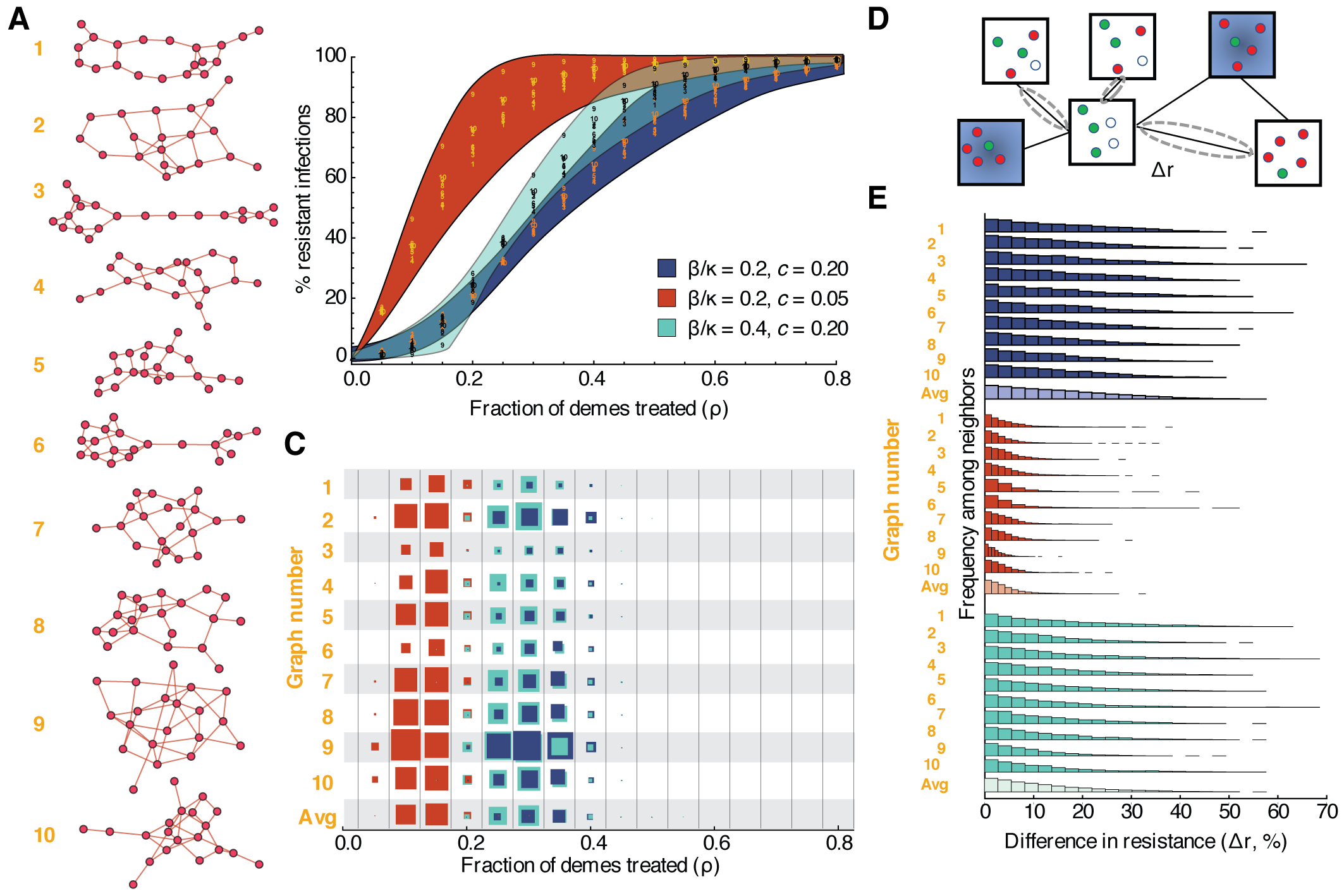

Supplement: S9 Fig — A) Randomly generated population structures on which infection was simulated. Each node represents a deme (a well-mixed sub-population of individuals), and each edge indicates that infection can spread in either direction between those two demes. Each network had twenty demes and the number of neighbors of each deme was drawn from a gamma distribution with mean three and a different variance. Ten example populations were selected out of 1000 total simulated to represent a broad range of variances in connectivity: from graph 1:4.4, 2:5.7, 3:6.2, 4:6.6, 5:6.9, 6:7.2, 7:7.5, 8:8.0, 9:8.6, 10:10.5. B) Fraction of infections that are resistant in the entire population (y-axis) versus fraction of demes treated, ρ (x-axis). Each color represents a different parameter set (blue background—baseline, red background—lower cost of resistance, teal background—more between-deme connectivity). Numbers show data points for the ten example populations. The colored envelope is created by shading between sigmoidal curves that encompass all the data. C) For each population structure shown (y-axis) and each treatment level (x-axis), the proportion of simulations that resulted in robust coexistence between drug-sensitive and drug-resistant strains is shown (by the colored area of the box). Robust coexistence was defined as at least 80% of demes supporting both strains at frequencies above 10%. D) Differences in resistance levels (% of all infections that are with the drug resistant-strain) are measured between all pairs of directly-connected untreated demes. E) Histograms showing the distribution of pairwise differences in resistance for a given population structure. Lighter shaded histograms combine results from all population graphs. All simulations used kinetic parameters κ = 0.25/day, g = 0.1/day, and ϵ = 0.9, and pooled results from 100 simulations with different random allocation of treatment across demes. Pairwise differences were calculated with 30% treatment. (TIF) [file pcbi.1008010.s010.tif]

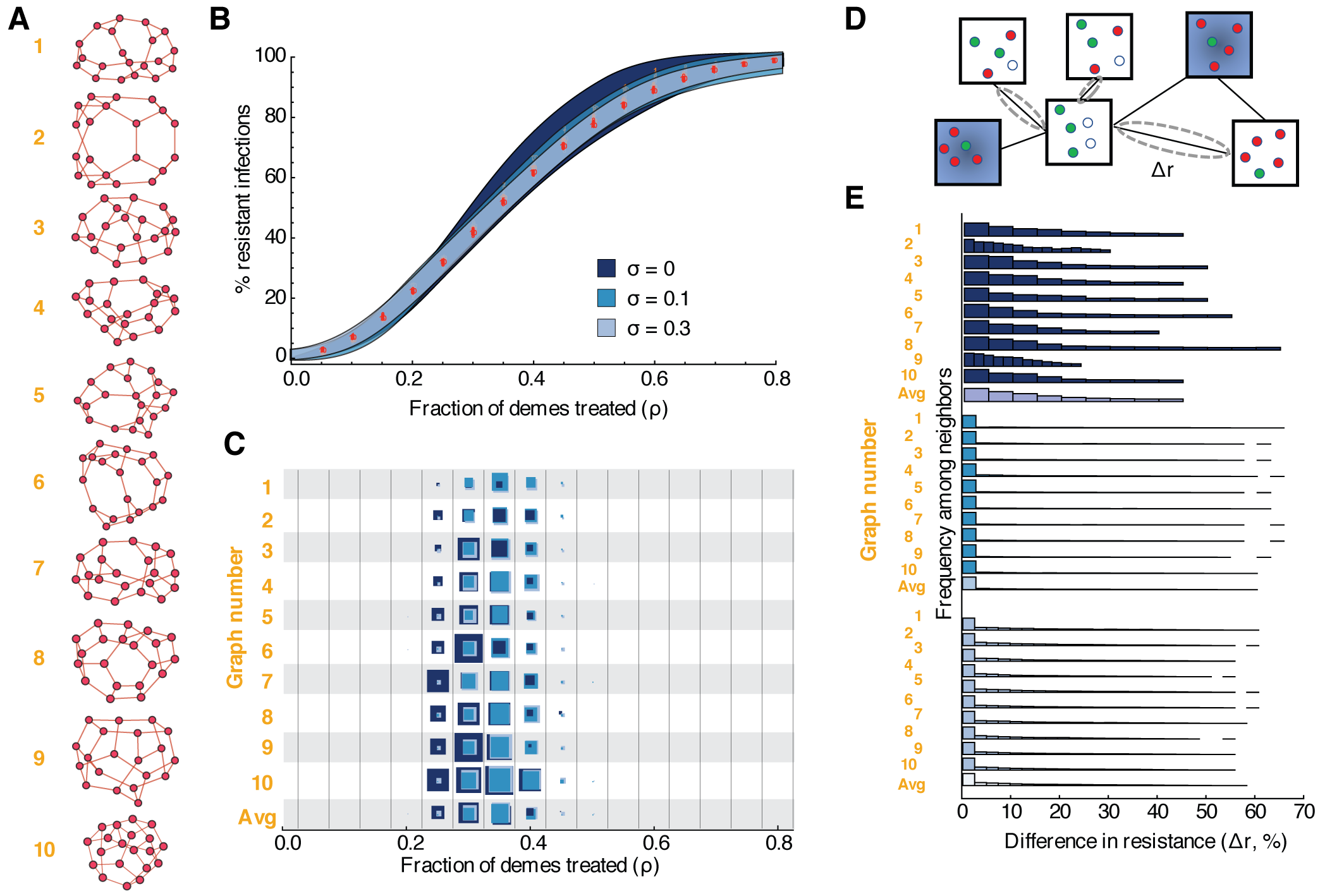

Supplement: S10 Fig — A) Randomly generated population structures on which infection was simulated. Each node represents a deme (a well-mixed sub-population of individuals), and each edge indicates that infection can spread in either direction between those two demes. Ten example populations were selected out of 1000 total simulated, each with twenty demes randomly connected to three neighbors each, to represent a broad range of outcomes. Deme sizes were chosen from a normal distribution with coefficient of variation of σ = 0, 0.1, of 0.3. B) Fraction of infections that are resistant in the entire population (y-axis) versus fraction of demes treated, ρ (x-axis). Each color represents a different level of heterogeneity in deme size (n navy blue—uniform sizes (σ = 0), medium blue—σ = 0.1, light blue—σ = 0.3). Numbers show data points for the ten example populations. The colored envelope is created by shading between sigmoidal curves that encompass all the data. C) For each population structure shown (y-axis) and each treatment level (x-axis), the proportion of simulations that resulted in robust coexistence between drug-sensitive and drug-resistant strains is shown (by the colored area of the box). Robust coexistence was defined as at least 80% of demes supporting both strains at frequencies above 10%. D) Differences in resistance levels (% of all infections that are with the drug resistant-strain) are measured between all pairs of directly-connected untreated demes. E) Histograms showing the distribution of pairwise differences in resistance for a given population structure. Lighter shaded histograms combine results from all population graphs. All simulations used kinetic parameters κ = 0.25/day, β = 0.05/day, g = 0.1/day, c = 0.2, and ϵ = 0.9, and pooled results from 100 simulations with different random allocation of deme sizes and treatment across demes. Pairwise differences were calculated with 30% treatment. (TIF) [file pcbi.1008010.s011.tif]

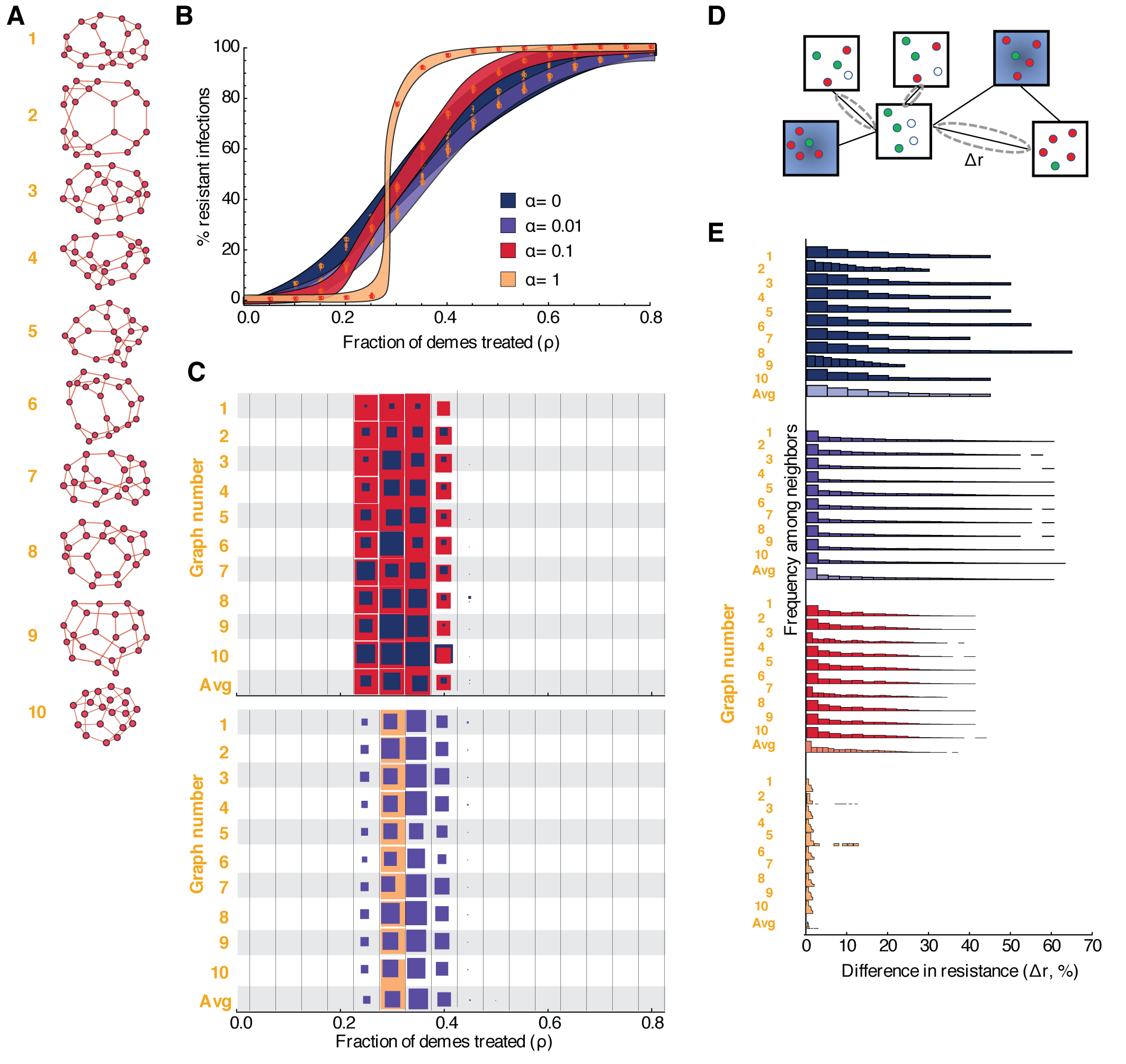

Supplement: S11 Fig — A) Randomly generated population structures on which infection was simulated. Each node represents a deme (a well-mixed sub-population of individuals), and each edge indicates that infection can spread in either direction between those two demes. Each population consisted of twenty demes randomly connected to three neighbors each, and additionally connected with relative weight α to all other demes. Ten example populations were selected out of 1000 total simulated to represent a broad range of outcomes. B) Fraction of infections that are resistant in the entire population (y-axis) versus fraction of demes treated, ρ (x-axis). Each color represents a different level of background connectivity between all demes (navy blue—no background mixing (α = 0), dark blue—α = 0.01, medium blue—α = 0.1, light blue—α = 1). Numbers show data points for the ten example populations. The colored envelope is created by shading between sigmoidal curves that encompass all the data. C) For each population structure shown (y-axis) and each treatment level (x-axis), the proportion of simulations that resulted in robust coexistence between drug-sensitive and drug-resistant strains is shown (by the colored area of the box). Robust coexistence was defined as at least 80% of demes supporting both strains at frequencies above 10%. D) Differences in resistance levels (% of all infections that are with the drug resistant-strain) are measured between all pairs of directly-connected untreated demes. E) Histograms showing the distribution of pairwise differences in resistance for a given population structure. Lighter shaded histograms combine results from all population graphs. All simulations used kinetic parameters κ = 0.25/day, β = 0.05/day, g = 0.1/day, c = 0.2, and ϵ = 0.9, and pooled results from 100 simulations with different random allocation of treatment across demes. Pairwise differences were calculated with 30% treatment. (TIF) [file pcbi.1008010.s012.tif]

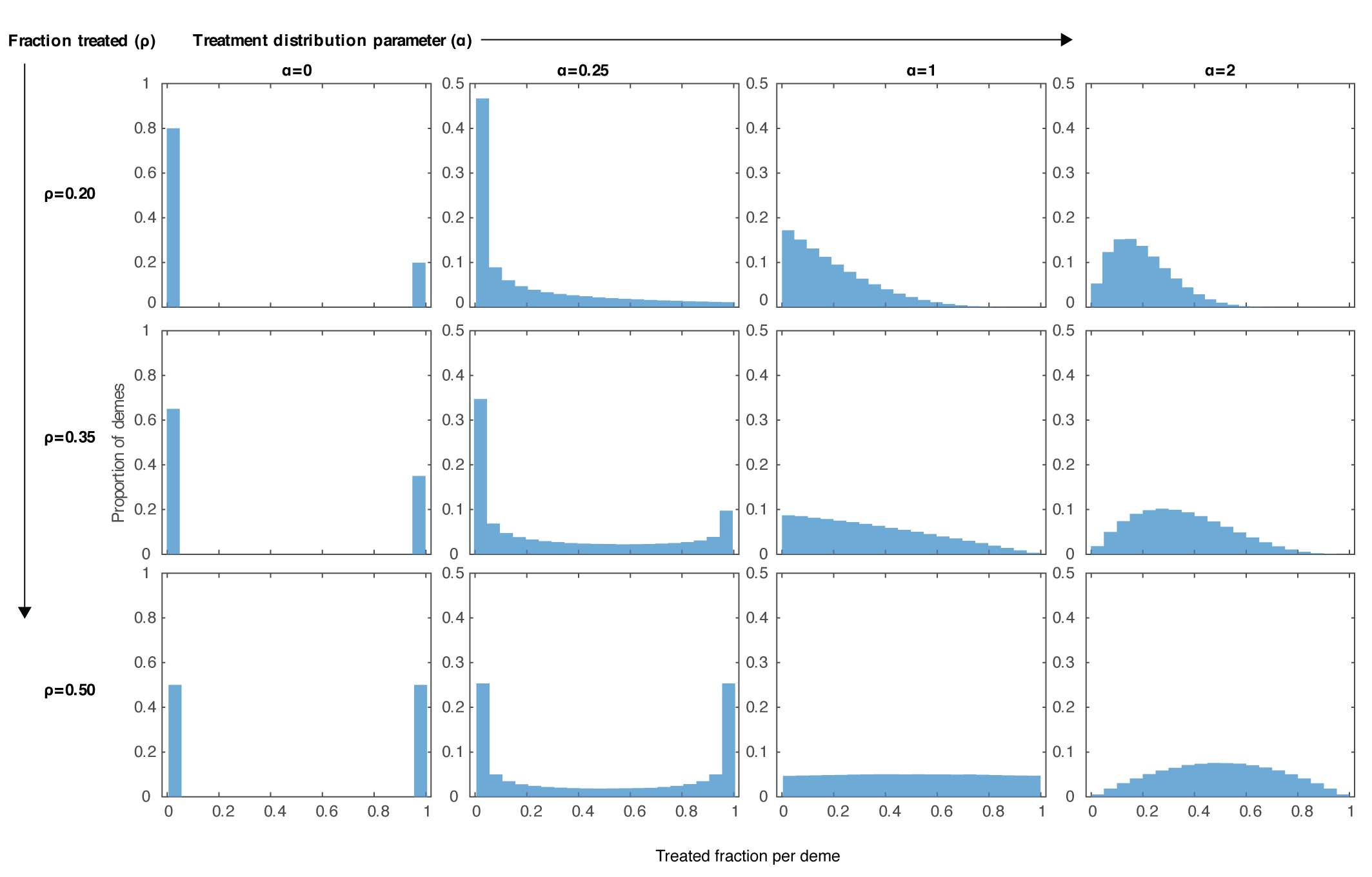

Supplement: S12 Fig — Each panel shows a distribution across demes of the fraction of individuals within the deme receiving treatment if infected. Treatment levels were drawn from Beta distributions with varying mean (ρ, rows) and shape parameter (α, columns). The left most column corresponds to the results presented in the rest of the paper, where individuals in each deme either have a 0 or 100% chance of being treated if infected. (TIF) [file pcbi.1008010.s013.tif]

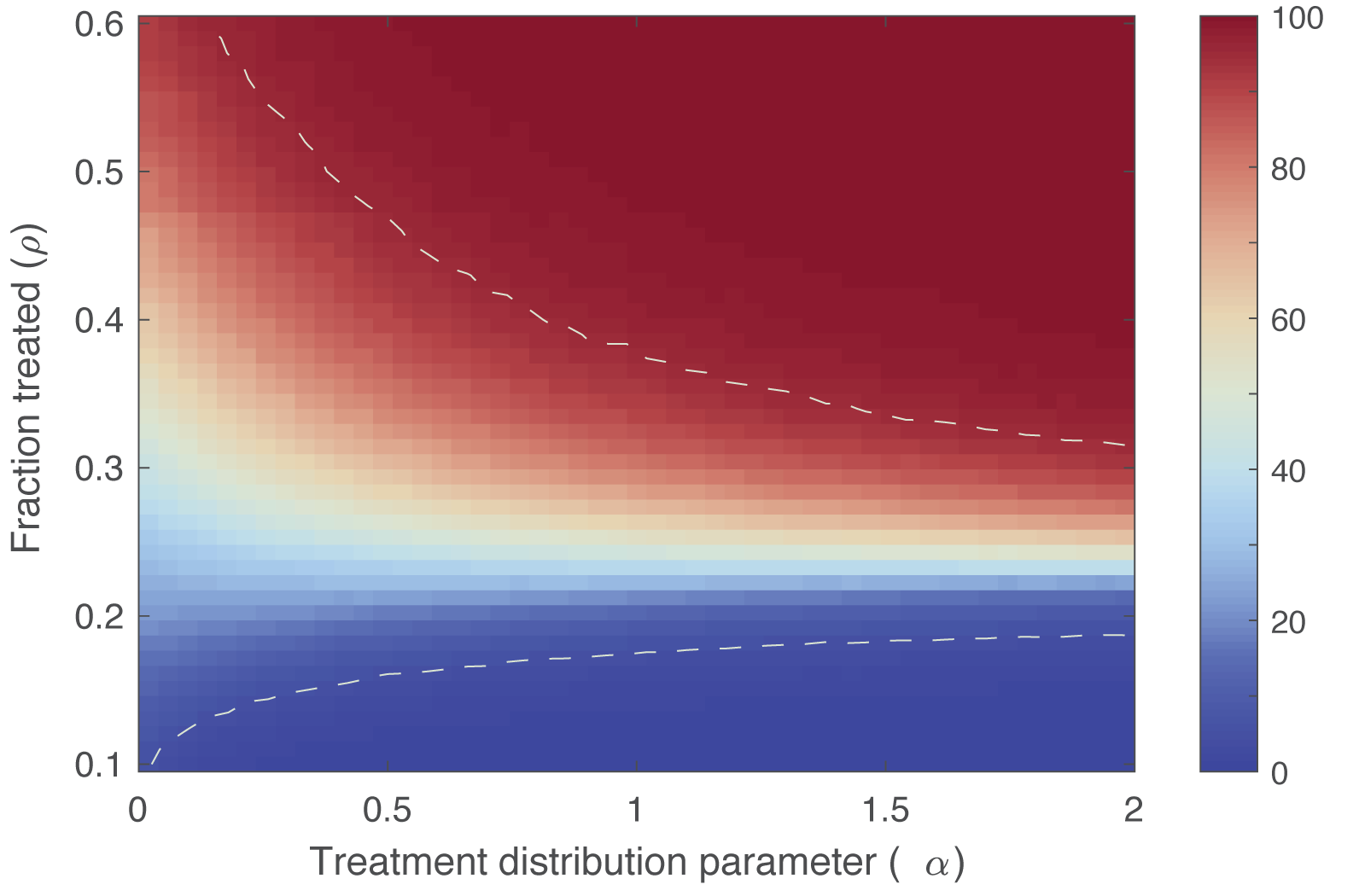

Supplement: S13 Fig — The percent of infections over the whole population that are caused by the the drug resistant strain as a function of the average treatment level across demes (ρ, y-axis) and the Beta-distribution parameter describing the spread of treatment levels (α, x-axis). Dotted lines border the region where resistance levels fall between 5% and 95%. Each panel shows a distribution across demes of the fraction of individuals within the deme receiving treatment if infected. Treatment levels were drawn from Beta distributions with varying mean (ρ, rows) and shape parameter (α, columns). Example treatment distributions are shown in S12 Fig. The left most edge of the graph (α = 0) corresponds to the results presented in the rest of the paper, where individuals in each deme either have a 0 or 100% chance of being treated if infected. Higher α values correspond to more continuous, unimodal treatment distributions with lower variance. The population consisted of twenty demes randomly connected to three neighbors each. Results were averaged over 1000 graphs with with 1000 random treatment allocations for each. For all results, the transmission rates are κ = 0.25/day (intra-deme) and β = 0.05/day (inter-deme), the recovery rate is g = 0.1/day, the cost of resistance is c = 0.1, and the treatment efficacy is ϵ = 0.9. (TIF) [file pcbi.1008010.s014.tif]

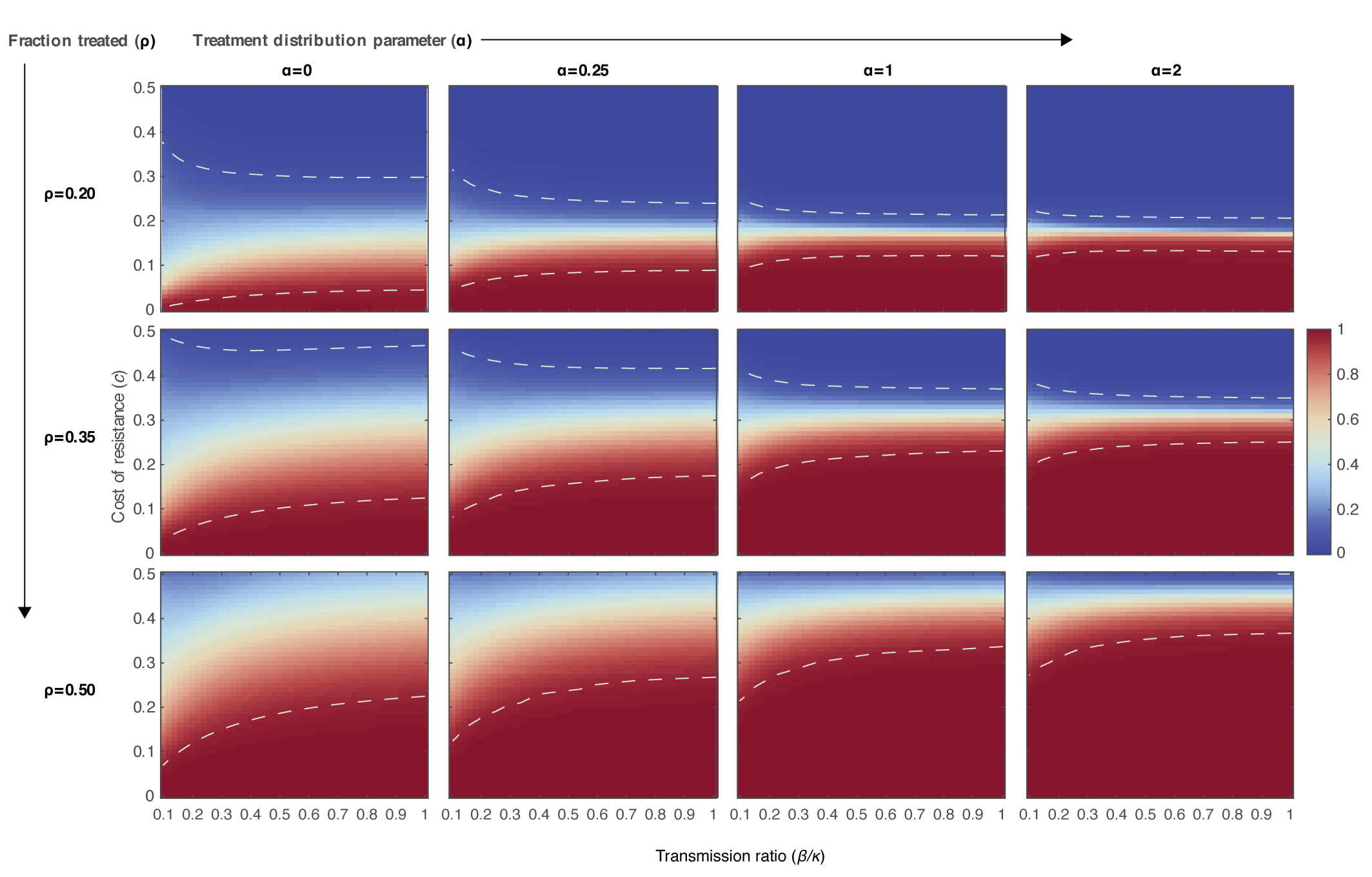

Supplement: S14 Fig — Each panel shows the average fraction of infections that are resistant in the entire population as a function of the relative connectivity between demes (β/κ) and the cost of resistance (c). Dotted lines border the region where resistance levels fall between 5% and 95%. The mean and variance in the amount of treatment per deme varied between panels and is given by the distributions in S12 Fig. The left most column corresponds to the case where individuals in each deme either have a 0 or 100% chance of being treated if infected. Higher α values correspond to more continuous, unimodal treatment distributions with lower variance. The population consisted of twenty demes randomly connected to three neighbors each. Results were averaged over 1000 graphs with with 1000 random treatment allocations for each. For all results, the intra-deme transmission rate is κ = 0.25/day, the recovery rate is g = 0.1/day, and the treatment efficacy is ϵ = 0.9. (TIF) [file pcbi.1008010.s015.tif]

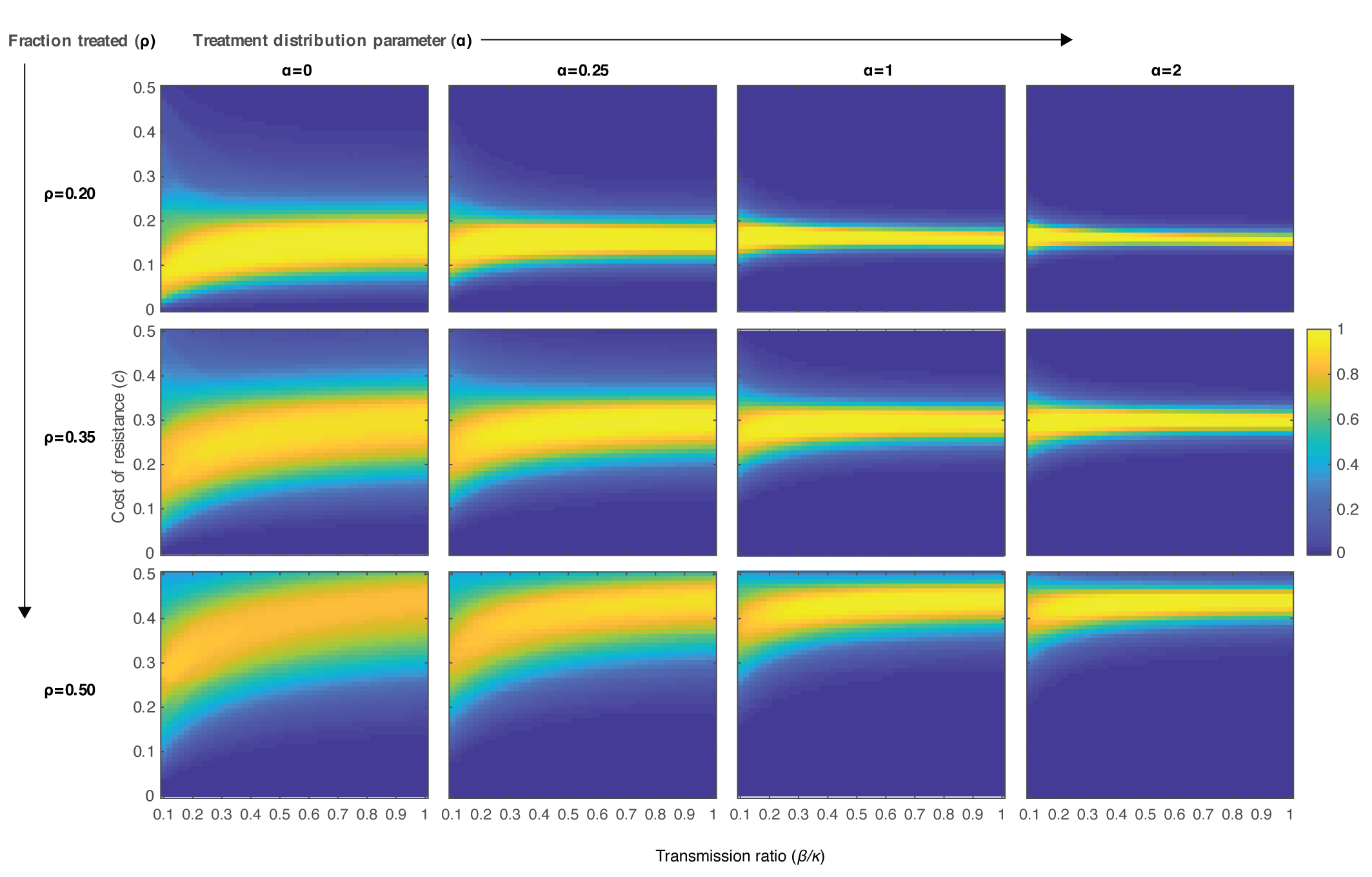

Supplement: S15 Fig — Each panel shows the average fraction of demes that supported both strains at frequencies of at least 10%, as a function of the relative connectivity between demes (β/κ) and the cost of resistance (c). The mean and variance in the amount of treatment per deme varied between panels and is given by the distributions in S12 Fig. The left most column corresponds to the case where individuals in each deme either have a 0 or 100% chance of being treated if infected. The population consisted of twenty demes randomly connected to three neighbors each. Results were averaged over 1000 graphs with with 1000 random treatment allocations for each. For all results, the intra-deme transmission rate is κ = 0.25/day, the recovery rate is g = 0.1/day, and the treatment efficacy is ϵ = 0.9. (TIF) [file pcbi.1008010.s016.tif]

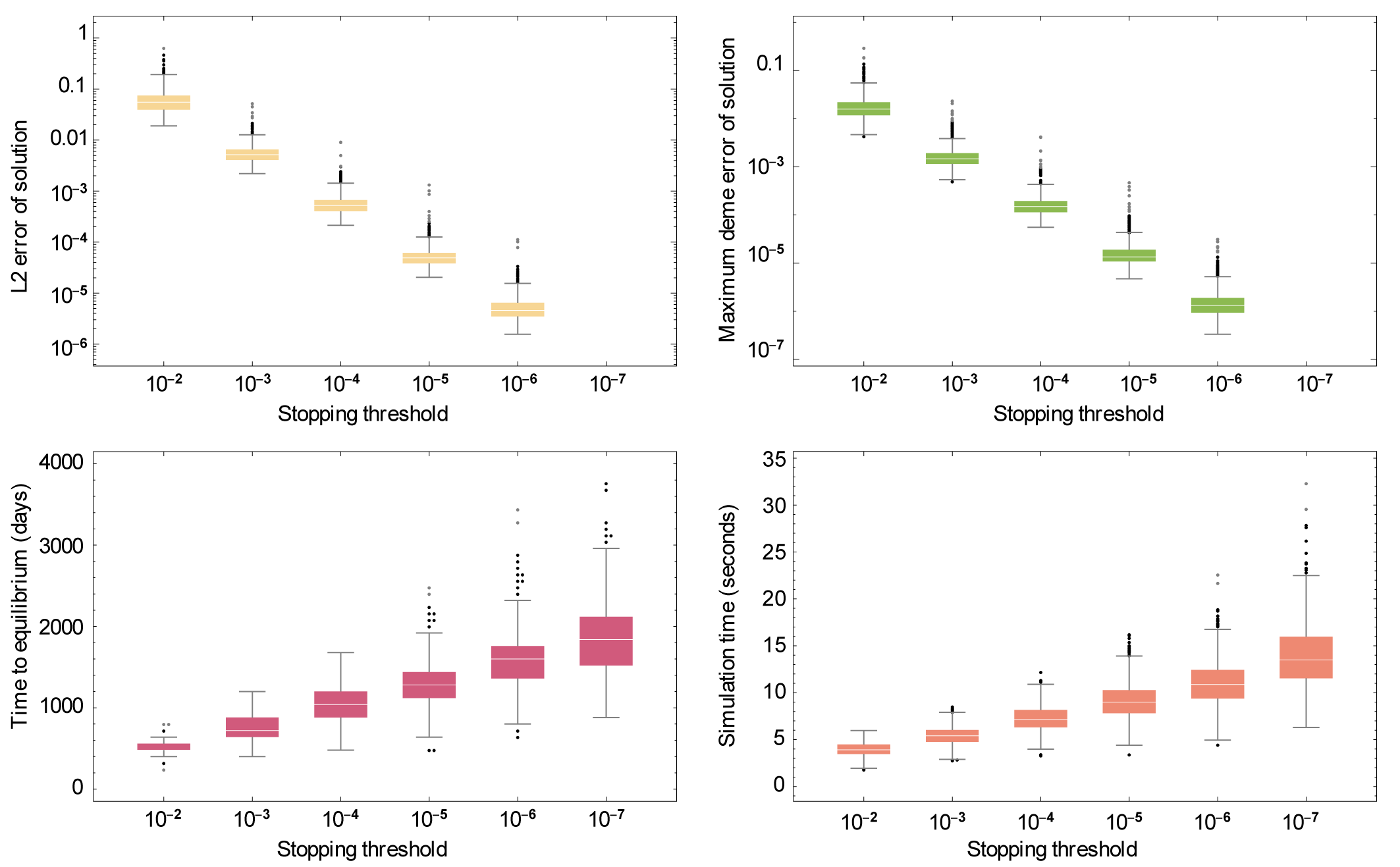

Supplement: S16 Fig — We tested the sensitivity of our results to the value of the derivative which we considered ``at equilibrium'', defined as the point where the sum of the derivatives of all strains in each deme was less than the stopping parameter divided by the number of demes. (Top row) We considered 10−7, the smallest value tested, as the ``true'' solution and measured this solution against other solutions with all parameters held equal, but the stopping parameter increased. Box plots show two representatives of the error from this solution as the stopping parameter is increased up to our parameter 10−4 used in the main-text results and beyond: (left), the matrix 2-norm (also known as L2 norm, Frobenius norm, or Euclidean distance). (Right): the maximum demewise error (L∞ norm). (Bottom row: For these same simulations, we show: (left), the time in internal units (simulated days) until equilibrium was reached; (right), the physical computation time of the simulation. The measures of error for our chosen parameter are small given the advancement in computation time afforded by that choice. (TIF) [file pcbi.1008010.s017.tif]

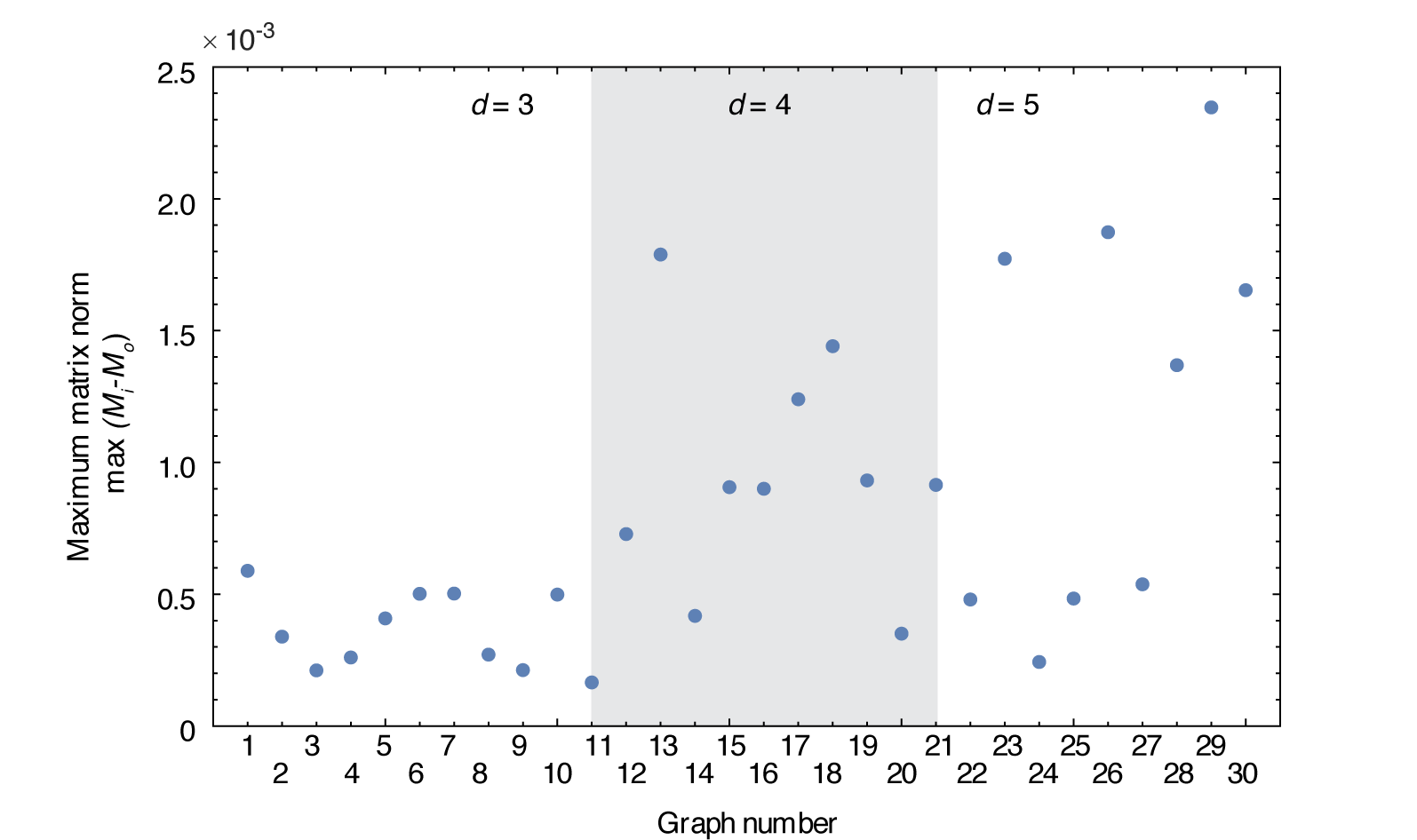

Supplement: S17 Fig — For 10 different random regular graphs with 20 demes and degree (d) 3, 4, or 5, treatment was allocated randomly across demes with an overall proportion treated ρ = 0.35. For each graph-treatment allocation combination, 100 different sets of initial conditions (levels of resistant and sensitive infections in each deme) were chosen uniformly at randomly, and infection dynamics were run until an equilibrium was reached as described in the Methods. The maximum difference (in matrix 2-norm, also known as L2 norm, Frobenius norm, or Euclidean distance) was calculated between the equilibria seen in any of 100 trials and the equilibrium values used for results reported in the main text. Very low maximum norm values suggest there is a single stable equilibrium value for each parameter set. (TIF) [file pcbi.1008010.s018.tif]
